# Generation and Characterization of Immortalized Mouse Cortical Astrocytes From Wildtype and Connexin43 Knockout Mice

**DOI:** 10.3389/fncel.2021.647109

**Published:** 2021-03-15

**Authors:** Antonio Cibelli, Sandra Veronica Lopez-Quintero, Sean Mccutcheon, Eliana Scemes, David C. Spray, Randy F. Stout, Sylvia O. Suadicani, Mia M. Thi, Marcia Urban-Maldonado

**Affiliations:** ^1^Dominick P. Purpura Department of Neuroscience, Albert Einstein College of Medicine, New York, NY, United States; ^2^Department of Medicine (Cardiology), Albert Einstein College of Medicine, New York, NY, United States; ^3^Department of Biomedical Sciences, New York Institute of Technology College of Osteopathic Medicine, New York, NY, United States; ^4^Department of Urology, Albert Einstein College of Medicine, New York, NY, United States; ^5^Department of Molecular Pharmacology, Albert Einstein College of Medicine, New York, NY, United States; ^6^Department of Orthopaedic Surgery, Albert Einstein College of Medicine, New York, NY, United States

**Keywords:** hTERT, Cx43 (connexin43), gap junction, cell model, astrocytes, immortalized astrocytes

## Abstract

We transduced mouse cortical astrocytes cultured from four litters of embryonic wildtype (WT) and connexin43 (Cx43) null mouse pups with lentiviral vector encoding hTERT and measured expression of astrocyte-specific markers up to passage 10 (p10). The immortalized cell lines thus generated (designated IWCA and IKOCA, respectively) expressed biomarkers consistent with those of neonatal astrocytes, including Cx43 from wildtype but not from Cx43-null mice, lack of Cx30, and presence of Cx26. AQP4, the water channel that is found in high abundance in astrocyte end-feet, was expressed at moderately high levels in early passages, and its mRNA and protein declined to low but still detectable levels by p10. The mRNA levels of the astrocyte biomarkers aldehyde dehydrogenase 1L1 (ALDH1L1), glutamine synthetase (GS) and glial fibrillary acidic protein (GFAP) remained relatively constant during successive passages. GS protein expression was maintained while GFAP declined with cell passaging but was still detectable at p10. Both mRNA and protein levels of glutamate transporter 1 (GLT-1) declined with passage number. Immunostaining at corresponding times was consistent with the data from Western blots and provided evidence that these proteins were expressed at appropriate intracellular locations. Consistent with our goal of generating immortalized cell lines in which Cx43 was either functionally expressed or absent, IWCA cells were found to be well coupled with respect to intercellular dye transfer and similar to primary astrocyte cultures in terms of time course of junction formation, electrical coupling strength and voltage sensitivity. Moreover, barrier function was enhanced in co-culture of the IWCA cell line with bEnd.3 microvascular endothelial cells. In addition, immunostaining revealed oblate endogenous Cx43 gap junction plaques in IWCA that were similar in appearance to those plaques obtained following transfection of IKOCA cells with fluorescent protein tagged Cx43. Re-expression of Cx43 in IKOCA cells allows experimental manipulation of connexins and live imaging of interactions between connexins and other proteins. We conclude that properties of these cell lines resemble those of primary cultured astrocytes, and they may provide useful tools in functional studies by facilitating genetic and pharmacological manipulations in the context of an astrocyte-appropriate cellular environment.

## Introduction

Vertebrate gap junctions (GJs) are formed by the connexin family of proteins. GJ channels provide direct cytosolic exchange of current-carrying ions, metabolic substrates and signaling molecules between adjacent cells. The most widespread and abundant GJ protein is connexin43 (Cx43), which is the primary GJ protein between cardiac myocytes, vasculature and astrocytes in the brain. Intercellular communication through Cx43 GJs mediates key essential functions in the tissues in which it is expressed, including propagation of contraction throughout the heart, signaling during migration of neural precursor cells, and coordination of cellular functions in communication compartments. Because it is such an abundant GJ protein, it was not surprising that when the first Cx43-null mouse was generated, the homozygous null pups died soon after birth (Reaume et al., 1995). What was surprising was that the perinatal mortality was not the result of disturbed rhythmic propagation throughout the heart, but instead was due to delayed neural crest cell migration and ensuing developmental defect of the left ventricular outflow tract (Lo and Wessels, 1998). As a consequence, the *foramen ovale* between the left and right cardiac chambers in Cx43-null mice does not close at birth. Thus, Cx43-null mice survive until placental circulation ends, at which time the animals are no longer adequately perfused.

In order to study the properties of cells lacking Cx43 expression, we maintained breeding colonies of Cx43 heterozygotes for many years so that we could obtain tissues and prepare primary cell cultures from wildtype and Cx43-null P0 pups immediately after delivery, or from E19-20 pups obtained by c-section (Scemes et al., 1998). This protocol has been costly and inefficient, as breeding is irregular in these transgenic mice, and cell cultures must be separately prepared from each individual mouse before genotype is verified. In order to circumvent these difficulties, we invested on generating immortalized astrocytes that would also be highly beneficial for allowing use of cultured astrocytes with, and without Cx43 expression for higher passage numbers, and comply with the three “Rs” for experimental animal model use (Reduce animal number, Refine techniques to minimize pain, and Replace animal experiments when possible) while saving time, money and reducing lab material waste. We transduced primary cultures of cortical astrocytes from Cx43-null and wildtype littermates with hTERT. This method has been successfully used to generate other cell lines, including wildtype and Cx43-null osteoblastic cell lines, as we previously described (Thi et al., 2010). In addition, hTERT transduction is reported to have the advantage over transfection with oncogenes in that it does not involve the inactivation of tumor suppressor genes (Lee et al., 2004).

In this manuscript, we report the characterization of hTERT-immortalized astrocyte cell lines derived from wildtype and Cx43-null mice, designated IWCA and immortalized Cx43-null cortical astrocytes (IKOCA) cells, with respect to expression of astrocyte-specific mRNAs and proteins as well as several diverse functional features. We also demonstrate that GJ plaques formed of Cx43 exogenously expressed in IKOCA cells are similar in appearance to GJ plaques expressed in the wildtype primary astrocyte cell culture, indicating the utility of these cell lines for studies of the impact of cellular and molecular manipulations on astrocyte structure and function.

## Materials and Methods

### Cell Cultures

We used primary cultures of cortical astrocytes derived from 19–20 day old (E19–20) wildtype (WT) and Cx43-null mouse embryos (offspring of Cx43 heterozygotes in C57Bl/6J-Gja1 strain, originally obtained from Jackson Laboratories). Animals were maintained at the Albert Einstein College of Medicine animal facility and all experimental procedures were approved by the college’s Institute of Animal Care and Use Committee and were performed using a modification of previously described astrocyte culture methods (McCarthy and de Vellis, 1980). Cortices were separated from whole brain E19-E20 embryos and, after removal of meninges, tissues were minced and enzymatically digested (0.05% trypsin at 37°C for 10 min). Cells from each animal were collected by centrifugation and pellets suspended in Astrocyte Medium [ScienCell Research Laboratory (SCRL), cat# 1801], supplemented with 2% fetal bovine serum (FBS) (SCRL cat# 0010), 1% penicillin/streptomycin (SCRL cat# 08030) and 1% astrocyte growth factor (SCRL cat# 1852), and seeded in plastic culture dishes and then shaken overnight at 37°C to remove non-astrocyte cells from the culture. Genotypes of the astrocyte culture obtained from each mouse pup were determined by PCR on tail DNA as described (Dermietzel et al., 2000). Astrocytes were maintained for 10–14 days in culture (100% humidity; 95% air/5% CO_2_, 37°C) until confluency, at which time cells were immortalized as we previously described for osteoblasts (Thi et al., 2010).

### Immortalization

We used the human telomerase reverse transcriptase (hTERT) overexpression system for immortalization of astrocytes. hTERT cDNA was PCR amplified from its original construct hTERT-pGRN145 (ATCC, MBA-141; Manassas, VA, United States) and subcloned into pLentiV5-EF1α vector (modified from original vector purchased from Invitrogen, cat# V49,610). Confluent cultures of WT and Cx43-null astrocytes were transduced with lentiviral particles containing hTERT cDNA 9 × 10^5^ transduction units (TU; particles/ml). After overnight incubation, the mixture was replaced with Astrocyte Medium. We used Astrocyte Growth Supplement (SCRL# 1,852). This supplement was chosen for our mouse cell culture because it was the commercially available supplement designed for astrocyte culture (human astrocytes specifically) and we intended to develop cell culture methods balanced for wide utility, convenience, and similarity to *in vivo* astrocytes. Selection of immortalized cells was then achieved by successive splitting bi-monthly, thus eradicating cells that did not continue to divide. Cells that were successfully transduced with hTERT viral particles were designated as immortalized beginning at passage two because primary astrocytes grow only very poorly beyond second passage. The immortalization process was performed four independent times in different litters to generate four cell lines of immortalized WT cortical astrocytes (IWCA) and four cell lines of immortalized Cx43-null cortical astrocytes (IKOCA).

### Quantitative Polymerase Chain Reaction

The quantitative polymerase chain reaction (qPCR) was performed using SYBR GREEN Master Mix (Applied Biosystems) in an Applied Biosystems 7,300 Real-Time PCR System (Forester City, CA, United States), according to the manufacturer’s instructions. Briefly, 2 μg total RNA was reverse transcribed into cDNA using SuperScript VILO cDNA Synthesis (Invitrogen). Amplification was carried out for 40 cycles with annealing temperature of 60°C in 25 μl final volume. Finally, a dissociation profile of the PCR product(s) was obtained by a temperature gradient running from 60°C to 95°C. The primers used for qPCR are listed in Table 1. Two technical replicas for each of the four immortalized WT and Cx43-null astrocyte cell lines were amplified. Relative gene expression levels of the analyzed mRNA samples were calculated using the ΔΔCt method, where values obtained for the gene of interest are first normalized to those of the reference, housekeeping gene, β-actin, and subsequently to those of their respective controls (non-hTERT-immortalized cells – passage 1). As in our previous studies (Thi et al., 2010), validation of the comparative Ct method was obtained after estimation of equality of efficiencies of target and reference amplifications [ΔCt (Ct_target_ – Ct_reference_)] over serial sample dilutions. Cells were retained from early passages and selected later passages as indicated for comparison of biomarker expression.

**TABLE 1:** qPCR primer sequences used for characterization of immortalized astrocytes.

Cx43 sense Mus/Rat	CCTCCTGGGTACAAGCTGGT	Marcia Maldonado
Cx43 antisense Mus/Rat	CGATTTTGCTCTGCGCTGTA	
βActin sense Mus/Rat	CGTTGACATCCGTAAAGACC	Marcia Maldonado
βActin Antisense Mus/Rat	TCTCCTTCTGCATCCTGTCA	
GFAP sense Mus	AGAAAACCGCATCACCATTC	Sarah Lutz
GFAP antisense Mus	TTGTGACTTTTTGGCCTTCC	
Cx26 sense Mus/Rat	GCCTACCGGAGACATGAAAA	Sarah Lutz
Cx26 antisense Mus/Rat	TACGGACCTTCTGGGTTTTG	
Cx30 sense Mus	CTTCATTTCGAGGCCAACTG	(Mylvaganam et al., 2010)
Cx30 antisense Mus	GGTAACACAACTCGGCCAC	
Glutamine Synthetase sense Mus	CAGGCTGCCATACCA ACTTCA	Marcia Maldonado
Glutamine Synthetase Antisense Mus	GAATGTGGTACTGGTGCC TCTTG	
APQ4 sense Mus	TGATGTTACTGGTTCAATAGC TTTAGC	(Rutkovskiy et al., 2012)
AQP4 antisense Mus	AAGATCGAGCTGGATTCA TGCT	
ALDH1L1 sense Mus	GGCTCTTCACCTGGCATCTTA	Marcia Maldonado
ALDH1L1 antisense Mus	GGCCCATAACCAGGGACAAT	
GLT1 sense Mus	TGGTCACCATGCTCCTCATT	Marcia Maldonado
GLT1 antisense Mus	CAAAAGAATCGCCCACCACA	

### Western Blot Analysis

Confluent astrocyte cultures plated in 100 mm dishes were lysed and equal amounts of total protein were loaded onto 7.5 or 10% SDS-PAGE gels for separation and electrophoretically transferred to nitrocellulose membranes (Whatman, Dassel, Germany). The membranes were probed with polyclonal antibodies to rabbit anti-Cx43, 1:10,000 (Sigma, cat# C6219); mouse anti-GFAP 1: 500 (Sigma cat# G3893), goat anti-GS 1:500 (Santa Cruz, cat# SC-6640), and goat anti-AQP4 1:500 (Santa Cruz, cat# SC-9888), followed by secondary antibody incubation with horseradish peroxidase-conjugated donkey anti-goat IgG, goat anti-rabbit IgG and goat anti-mouse (1:10,000, Santa Cruz Biotechnology, Santa Cruz, CA, United States cat# SC-2020, SC-2004, and SC-2005, respectively). Protein bands were detected using the Immobilon Western Chemiluminescent HRP Substrate (EMD Millipore, MA, United States catalog# WBKLS0100) and images were acquired using the *In Vivo* FX PRO imaging system (Carestream, Carestream, NY, United States).

### Dye Coupling

We used both dye injection of Lucifer Yellow (LY) and the so-called “parachute assay” with calcein-AM loaded donor cells to evaluate GJ-mediated intercellular diffusion of moderately large molecules (LY = 444 Da; calcein = 622 Da). Dye-injection was performed in astrocyte cultures of similar confluency. LY was iontophoresed (continuous current of 0.1 μA) for 5 min using an electrometer (model 3100; A-M Systems) into single cells using sharp microelectrodes filled with the dye (LY, 5% wt in 150 mM LiCl; resistance ∼100 MOhms). After removal of the micropipette, images were immediately acquired using a CoolSNAP-HQ2 CCD camera (Photometrics, Tucson, AZ, United States) attached to a Nikon inverted microscope with 10X dry objective (numerical aperture 0.3) and FITC filter sets. Fluorescent cells surrounding the LY-injected cell were counted, and the values were then normalized by the total number of cells within the injected region of interest (ROI) and expressed as percentage of coupled cells per ROI. The parachute assay was performed essentially as described (Stains and Civitelli, 2016; McCutcheon et al., 2020a). In brief, cells in culture were detached via trypsinization (Thermo Fisher Scientific, #25200056), loaded in suspension with 10 μM calcein-AM (Thermo Fisher Scientific, #C3100MP) and labeled with the lipophilic GJ impermeable dye DiI (Sigma Aldrich, #468495-100MG) for 30 min to distinguish the parachute donor cells from the recipient cells. Donor cells were then rinsed with Dulbecco’s phosphate buffer saline (DPBS, Mediatech, Cellgro, VA, United States) and centrifuged twice to remove residual dye, then added to confluent monolayers of unlabeled recipient cells. Number of recipient cells adjacent to the donor cells stained with GJ permeant calcein dye was determined for paired IWCA-IWCA, IKOCA-IKOCA, and heterocellular IWCA-IKOCA. This assay is both confirmatory and supplemental to the LY injections, as it depends both on rate of GJ formation and on permeability of the GJs that are formed.

### Electrical Coupling

The dual whole-cell voltage-clamp technique was used to characterize junctional conductance in IWCA and IKOCA cells, as described (del Corsso et al., 2006). Pairs of astrocytes were voltage-clamped at holding potentials of 0 mV, and 8–10 s duration command steps (ΔV) in 20 mV increments from −110 to +110 mV or from −100 to +100 mV were presented to one cell with pClamp software (Axon Instruments, Foster City, CA, United States). In some experiments a voltage ramp protocol was used, in which voltage slowly increased from −100 to +100 mV. Junctional currents (Ij) were recorded in the unstepped cell, and junctional conductance (Gj) was calculated as −Ij/ΔV (del Corsso et al., 2006). Patch pipettes were filled with (in mM) 140 CsCl, 10 EGTA, and 5 Mg_2_ATP, pH 7.25. Cells were bathed in solution containing (in mM) 140 NaCl, 2 KCl, 2 CaCl_2_, 1 BaCl_2_, 2 CsCl, 1 MgCl_2_, and 5 HEPES, pH 7.2.

### Transient Exogenous Expression of Fluorescent Protein-Tagged Cx43

Immortalized Cx43-null cortical astrocytes cells were transfected with superfolderGFP-tagged Cx43, msfGFP-rCx43, EBFP2-rCx43, sfGFP-Cx30, sfGFP-Cx26 (Stout et al., 2015; Stout and Spray, 2016), and msfGFP-ATG9A. This recovered Cx43 expression to allow comparison of exogenously expressed Cx43 aggregates to endogenously expressed plaques in primary WT astrocyte cultures. Cells were plated to 50–60% confluence in DMEM (Thermo Fisher Scientific, #11885084) supplemented with 10% FBS and 1% penicillin/streptomycin (Thermo Fisher Scientific, #15070063). Two hours prior to transfection, the media was changed. For transfections, 1 μg DNA was combined with 100 μL Opti-MEM (Thermo Fisher, #31985062) and 5 μL transfection reagent (Mirus TransIT LT1, #MIR2300) and incubated at room temperature for 20–30 min. Liposome-DNA complexes were then added dropwise to IKOCA monolayers and incubated in standard culture conditions without changing media for 2–4 days before imaging.

### Confocal Microscopy to Analyze Distributions of Astrocyte Proteins and to Compare Endogenous Cx43 Junctions With Those Formed by Fluorescent Protein-Tagged Cx43

To evaluate protein localization within the WT and Cx43-null primary astrocytes and immortalized cell lines, cultures plated on coverslips were fixed with 4% paraformaldehyde (PFA), permeabilized with 0.4% Triton-X100, and blocked with 2% donkey serum (Jackson Immunoresearch, West Grove, PA, United States) as previously described (Scemes et al., 2000). The cells were then incubated with primary antibody against rabbit anti-Cx43, 1:1,000 (Sigma, cat# C6219); mouse anti-GFAP 1: 500 (Sigma cat# G3893), goat anti-GS 1:500 (Santa Cruz, cat# SC-6640), and goat anti-AQP4 1:500 (Santa Cruz, cat# SC-9888), and secondary antibody conjugated to Alexa-488 goat-anti-mouse IgG (cat#A11001), Alexa Fluor 594 Donkey Anti-goat (cat#A-11058) and Alexa-488 goat anti-rabbit IgG (cat#A-11034) (1:1000, Invitrogen). The coverslips were mounted on slides, examined on a Zeiss LSM 510 DUO Laser Scanning Confocal Microscope (Carl Zeiss) with a 40× water-immersion objective. Stacked images were taken serially at 0.6 μm *z* axis steps.

Cx43 was either immunolabelled with primary rabbit polyclonal antibody (1:1000; Abcam, #ab11370) in permeabilized cells, as described above, or was identified by the fluorescent signal of GFP-tagged Cx43 (green fluorescent protein) in transfected cells. Image acquisition and z-stacks of immunolabeled junctional plaques were obtained with Zeiss LSM 880 inverted microscope with a 63× oil objective (numerical aperture, 1.4). *Z*-stacks of GJ plaques of exogenously expressed GFP-tagged Cx43 in live cells were obtained at 37°C with Zeiss 5Live Duo with 63× oil immersion objective. Imaging media for live cells was phenol-free DMEM (Thermo Fisher Scientific, #31053028) supplemented with 10% FBS, 1% PS, and 25 mM HEPES (Thermo Fisher Scientific, #15630080).

### Imaging of IKOCA Cells Transfected to Express Fluorescent Protein-Tagged Connexins With co-Staining via Immunofluorescence

Immortalized Cx43-null cortical astrocytes cells were grown in Ibidi 8-well chambered ibiditreat chambered slides (Ibidi Inc.) and transfected with EBFP2-Cx43 then than 48 h after transfection the were fixed in 4% PFA for 10 min, permeabilized with 0.4% Triton-X100, and blocked with 2% donkey serum and then immunostained using rabbit antibody against Cx43 (Figure 6A, Sigma, cat# C6219, 1:500) or Alpaca antibody against Green Fluorescent Protein conjugated to Alexa Fluor 488 (also labels EBFP2, Figure 6B, Chromotek, cat# gb2AF488, 1:100 dilution) to enhance the EBFP2 signal in the green color channel along with DAPI (blue to stain nuclei). To co-label mitochondria we stained with TOMM20 primary antibody (Invitrogen, cat# MA5-32148, 1:500) followed by secondary staining with Donkey anti-rabbit Alexa Fluor 647 (Figure 6B, Invitrogen, cat# A-31573, 1:500). We stained the cells in Figure 6A with Alexa 488 goat anti-rabbit IgG (Invitrogen, cat# A-11034, 1:500). Primary antibody labeling was performed for 1 h at room temperature with agitation then overnight at 4 degrees. Secondary antibody staining was done for 3 h at room temperature with agitation. Images in Figure 6 were captured using a Zeiss 980 Airyscan2 laser scanning microscope with a 63 × 1.4NA Plan-Apochromat oil immersion objective. The zoomed-out image in Figure 6B has a non-linear intensity lookup table for the green channel to allow the reader to simultaneously see both the very densely labeled reflexive GJ plaque and the non-junctional membrane localized Cx43. All other image lookup tables are scaled linearly by signal intensity.

### Live Imaging and Fluorescent Recovery After Photobleach Experiments on IKOCA Cells Expressing Fluorescent Protein-Tagged Proteins via Transient Transfection

Immortalized Cx43-null cortical astrocytes cells were grown in Ibidi 8-well chambered slides transfected as described for previous sections. sfGFP-Cx43, sfGFP-Cx30, and sfGFP-Cx26 were used as described previously for other cell types (Stout et al., 2015). Cells were transferred to imaging media containing 10% FBS, 25 mm HEPES, and 2 mm glutamine in DMEM without phenol red. Live experiments in Figure 7 were carried out on the Zeiss 5live Duoscan 510 confocal laser scanning microscope with the 63 × 1.4 NA objective. 3D FRAP experiments were carried out by centering an 11 plane *Z*-stack acquisition on a GJ plaque then acquiring an 11-plane 3D image every 3 s. A stripe photobleach was performed with the 488 nm laser at 100% with three iterations. The resulting 3D-timelapse image was converted to a maximum projection orthogonal reconstruction to produce a single-plane time-lapse image series in Figure 7A. For Figure 7B, IKOCA cells were co-transfected with EBFP2-Cx43 (McCutcheon et al., 2020b) along with human ATG9A tagged on the amino-terminus with monomeric superfolder GFP (msfGFP) produced by standard subcloning techniques, with plasmid sequences available on request. Cells were then imaged with 11 plane Z-stack acquisitions with both blue and green channels sequentially imaged per focal plane. The resulting two-color, 3D time-lapse was collapsed to a time-lapse sequence of maximum projection orthogonal projections for display in Figure 7.

### Measurement of Barrier Formation in IWCA-Endothelial Co-Cultures

Transwell polyester inserts with 0.4 μm pores (Sigma Aldrich, #CLS3460) were flipped up-side down and coated with 100 μL of 30 μg/mL fibronectin (FN; Sigma Aldrich, #F1141) solution in DPBS. Coated inserts were incubated for 1 h at 37°C in a humidified incubator with 95% air/5% CO_2_. IWCA were then seeded at a density of 30,000 cells/cm^2^ and cells allowed to adhere to the FN coated Transwell insert for 2 h, after which the inserts were flipped again and placed in 12 multi-well plates, with the IWCA cells now facing the bottom of the plate, and incubated for 2 days in DMEM. Mouse brain endothelial cells (bEnd.3 cells, ATCC CRL-2299, Manassas, VA, United States) were then seeded on the luminal side of the insert at a density of 60,000 cells/cm^2^ and grown in DMEM. Companion inserts without IWCA were seeded with bEnd.3 alone to compare their barrier function to that of the co-cultures. Trans-endothelial electrical resistance (TEER) of endothelial monolayers and co-cultures of endothelial monolayers with IWCA was measured using the cellZscope system (Nanoanalytics, Munster, Germany) for automated, long-term monitoring of barrier resistance. Each insert seeded with cells was placed in a chamber filled with media (DMEM – 10% FBS), and the chamber holder was moved to the incubator (37°C, 5% CO_2_) and connected to the controller. CellZscope software measured TEER every 6 h. Media in the chamber and the inserts were replaced every 3 to 4 days and TEER was monitored for up to 7 days after seeding the bEnd.3 cells on the insert.

### Statistical Analysis

GraphPad Prism 8 and 9.0.1 software was used for data and statistical analyses. Data are represented as mean ± SEM and considered statistically significant at ^∗^*P* < 0.05. Statistical differences were determined by unpaired Student’s *t*-test for dye injection analyses. One-way ANOVA followed by Dunnett’s multiple comparison test was used for Western blot analysis and parachute dye transfer assay. Mixed-effects analysis was used to compare TEER between groups at each timepoint.

## Results

### Quantification of Expression Levels of Astrocyte Markers in Primary Cultures of WT and Cx43-Null Astrocytes, and at the Passages After Each Genotype Was Transfected With hTERT

**FIGURE 1:**
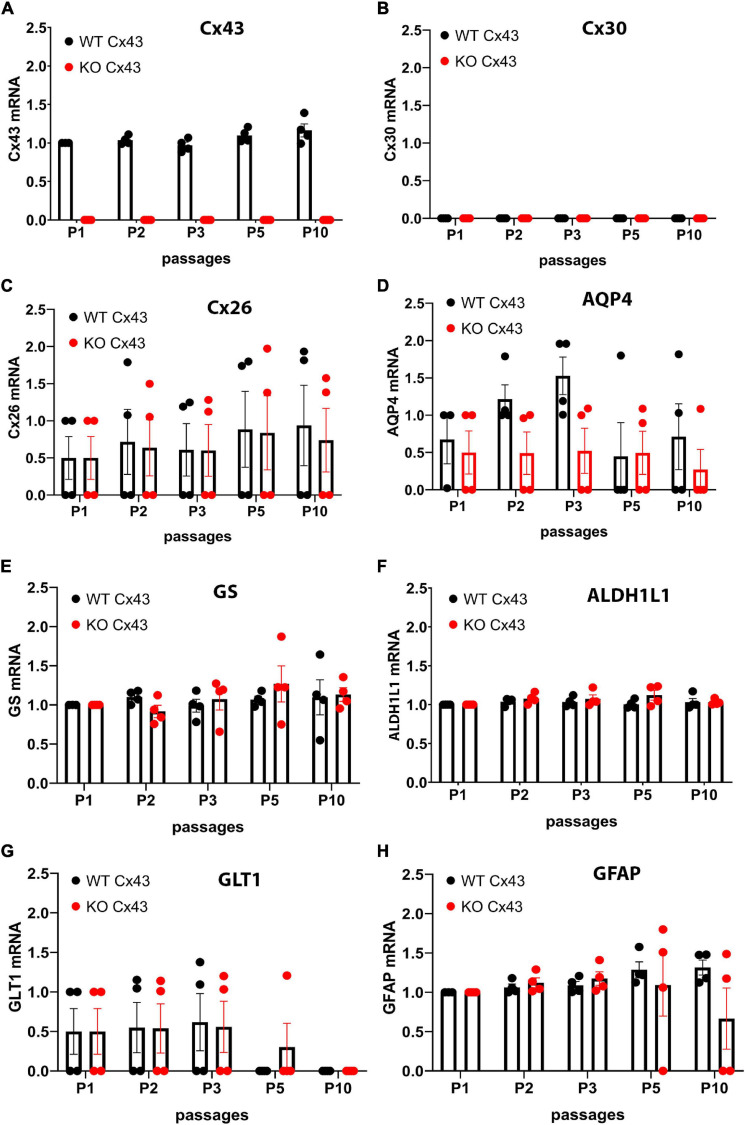
Quantitative RT-PCR showing expression levels of mRNAs encoding astrocyte proteins during ten passages after transduction. Expression levels of astrocyte biomarkers in the immortalized wildtype (IWCA) and Cx43-null (IKOCA) cortical astrocyte cell lines. Expression levels measured at each time point up to 10 passages after transduction with hTERT are normalized to those of IWCA astrocytes before transduction (labeled passage 1 in the histograms). Points in histograms correspond to individual cell lines and bars and variance correspond to mean ± SE values from the four independent clones generated for each of the two genotypes. **(A)** Cx43 levels are stable in IWCA up to 10 passages, whereas Cx43 expression is absent in all of the IKOCA lines. **(B)** Cx30 was not detectable in either IWCA or IKOCA, either before immortalization or at any time point thereafter. **(C)** Cx26 is present in both IWCA and IKOCA and is relatively stable in both genotypes with continued passaging. **(D)** Levels of AQP4 transcript in both IWCA and IKOCA, with the lowest levels at passage 10 being about 20% that of those in primary cells. **(E,F)** Transcript levels of the astrocyte markers glutamine synthetase (GS) and aldehyde dehydrogenase (ALDH1L1) were remarkably stable over the 10 passages examined. **(G)** mRNA for the glutamate transporter (GLT1) were lower than in primary cells but similar between genotypes and declined after passage 5 in both genotypes to reach undetectable levels at passage 10. **(H)** Levels of the astrocyte intermediate filament transcript GFAP was stable in both genotypes but was low in two of the IKOCA cell lines at passage 10.

Cx43 transcript expression was found to be relatively constant over time in IWCA astrocytes and was not at all detectable in IKOCA nulls (Figure 1A). Cx30 levels were too low to be quantified in both genotypes at all passages (Figure 1B), whereas Cx26 abundance was relatively constant in both genotypes up to passage 10 (Figure 1C). Aquaporin 4 (AQP4) mRNA showed similarly sustained expression, although levels were highly variable in different cell lines (Figure 1D). Other astrocyte markers that we examined included glutamine synthetase (GS), aldehyde dehydrogenase 1 (10-formyltetrahydrofolate dehydrogenase, ALDH1L1), glutamate transporter 1 [GLT-1, mouse ortholog of human excitatory amino acid transporter 2 (EAAT2) also known as solute carrier family 1 member 2 (SLC1A2)] and glial fibrillary acidic protein (GFAP). GS, ALDH1L1 and GFAP were found to have virtually constant mRNA expression levels over time in culture in both genotypes (Figures 1E,F,H). Expression of GLT-1 was present in two of the cell lines of both genotypes and below detection in the other 2 cell lines (Figure 1G).

**FIGURE 2:**
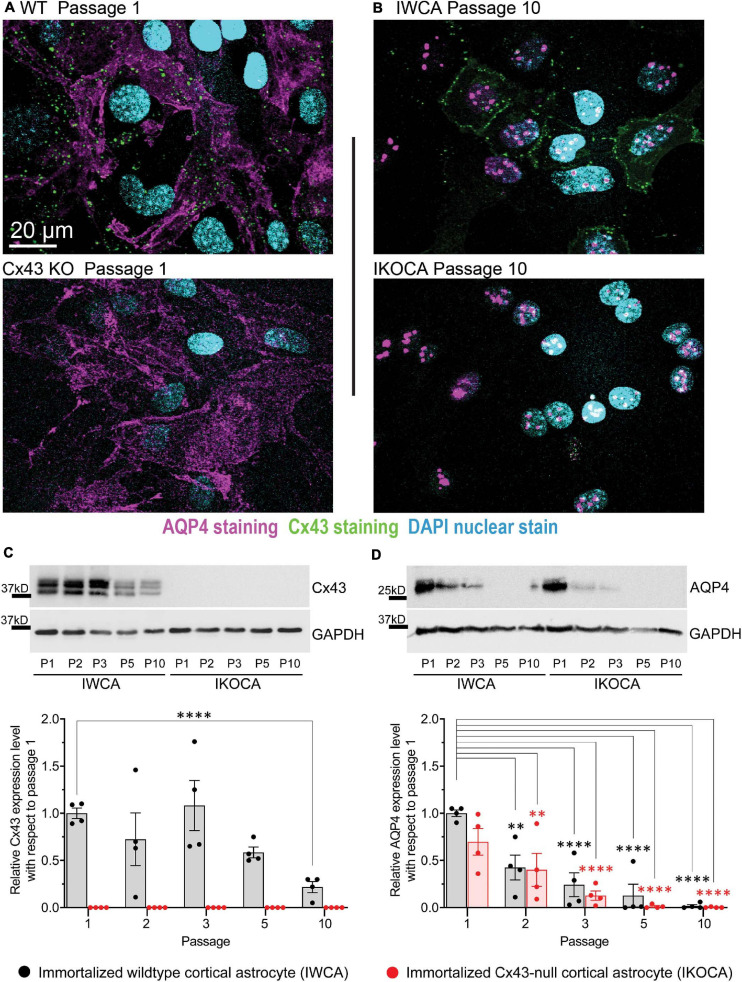
Expression level and localization of Cx43 and AQP4 in IWCA and IKOCA during the first ten passages. **(A,B)** Immunostaining of Cx43 in immortalized WT (IWCA) and Cx43-null (IKOCA) cortical astrocyte cultures revealed punctate intercellular localization in IWCA that was less abundant but still prominent over time in culture. IKOCA showed no Cx43 immunostaining at any time point. Punctate distribution of AQP4 indicated that this protein was still well expressed at passage 10 in both IWCA and IKOCA, but at reduced to very low levels. Note: dot like staining in the nuclei staining is non-specific, likely as artifact of AQP4-C19 antibody, as described previously (Thi et al., 2008). Scale bar is 20 μm and scale is the same for all microscope images in this figure. **(C)** Western blots showed no significant decline in Cx43 protein levels in IWCA until passage 10, when its level was reduced to about 30% of that of primary astrocytes. Cx43 protein expression was absent in IKOCA at all-time points. **(D)** AQP4 protein expression was very low but detectable at passage 5 and beyond in both genotypes.

Western blots and immunostaining were performed to quantify protein expression levels and determine cellular localization in the IWCA and IKOCA at passages 1–10. Immunoblots and immunostaining for Cx43 at representative passages are shown in Figures 2A–C, and for AQP4 in Figures 2A–D. Cx43 protein level was not significantly reduced until passage 5 in the IWCA cultures but was reduced to about 30% of its initial level at passage 10 (Figure 2C). Cx43 was not detected at any time point in immunostained cultures or in immunoblots obtained from IKOCAs. Both immunostaining and Western blots revealed that levels of AQP4 protein were lower in IKOCA than IWCA astrocytes at passage 1, and declined progressively in both genotypes from passage 1 onward; at passage 5 and beyond, basal AQP4 expression was quite low (Figure 2D).

**FIGURE 3:**
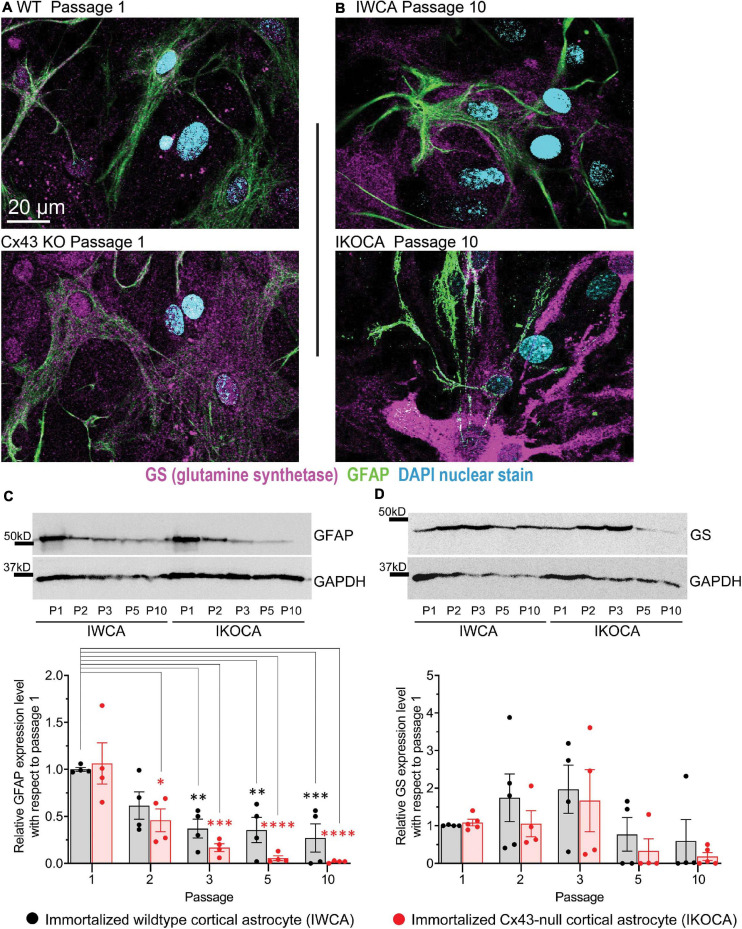
Expression level and localization of GFAP and GS in IWCA and IKOCA during the first ten passages. **(A,B)** GFAP immunostaining declined with time in culture for both IWCA and IKOCA. GS was highly expressed in both IWCA and IKOCA cultures at all passages but declined at passage 5 in both genotypes. Scale bar is 20 μm and scale is the same for all microscope images in this figure. **(C)** GFAP protein levels also declined rapidly with progressive passage, with minimal levels of about 27% in IWCA and about 2% in IKOCA cultures. **(D)** GS protein levels increased over time in culture, being somewhat variable at all-time points.

Expression levels and cellular localization were also determined for GFAP and GS, two other characteristic astrocyte cell markers. Similar to mRNA results shown in Figure 1, GFAP staining was detectable in both IWCA and IKOCA at all passages (Figures 3A,B), but protein levels declined over time (Figure 3C). However, this decline in GFAP protein expression was more rapid in the IKOCA than in IWCA, as revealed by the immunoblots (Figure 3C). GS showed strong immunostaining in both IWCA and IKOCA until passage 10 (Figures 3A,B). Western immunoblots detected an apparent increase in GS seen in both IWCA and IKOCA cultures until passage 5, which decreased thereafter (Figure 3D). Variance was high, and differences between genotypes or over time were not significant as evaluated by ANOVA.

### Assessment of Cx43 Function in the Immortalized Cortical Astrocyte Cell Lines

**FIGURE 4:**
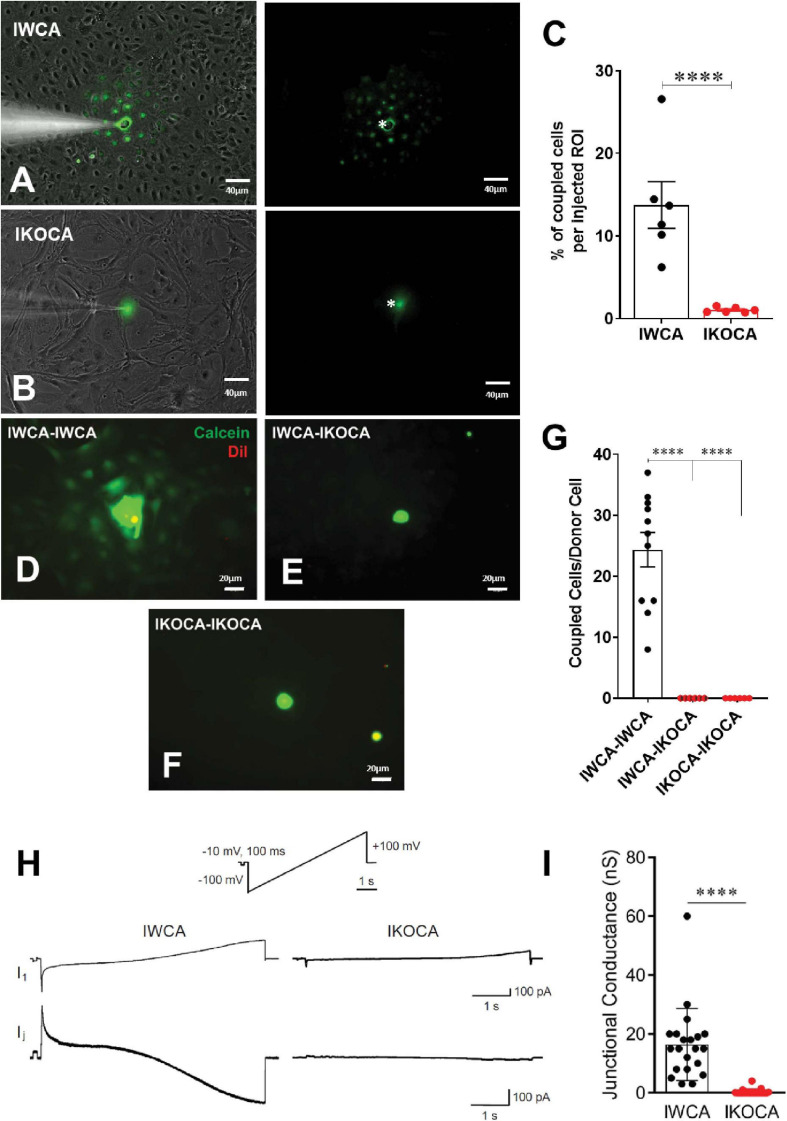
Assessment of functional GJ coupling in immortalized WT (IWCA) and Cx43-null (IKOCA) in confluent cortical astrocyte cultures. **(A)** Lucifer Yellow (LY) dye coupling between IWCA observed at passage 10. Left, bright phase contrast image of the field of view, with superimposed fluorescence. Right; fluorescence image with asterisk indicating the cell that was micro-injected with LY; note gap junction-mediated dye spread detected in all immediately adjacent neighbors. Scale bars = 40 μm **(B).** LY dye coupling was not observed between IKOCA at passage 10. Left, bright phase plus fluorescence showing field of view for injection. Right: In IKOCA cultures, the dye did not spread from the injected cell into the neighbor adjacent cells. Scale bars = 40 μm **(C)**. Quantification of LY dye spread in IWCA and in IKOCA cultures. Bar histograms correspond to mean ± SEM of six injections per genotype performed in two independent cultures per genotype at passage 10 (^****^p < 0.001). **(D–F)**. Parachute assay: **(D)** from IWCA passage 9 donor to recipient IWCA cells (IWCA-IWCA); **(E)** from IWCA donor to recipient IKOCA passage 10 cells (IWCA-IKOCA), and **(F)** from IKOCA passage 10 donor to recipient IKOCA cells. Donor cells were identified by double calcein-DiI (green and red, respectively) labeling. Scale bars = 20 μm **(G)** Quantification of calcein dye spread in IWCA-IWCA, in IWCA-IKOCA and in IKOCA-IKOCA donor-recipient cultures. Coupling was assessed as number of coupled cells per donor cell. **(H)** Junctional currents measured using dual whole cell voltage clamp between IWCA and IKOCA cell pairs. Slow voltage ramp (inset, –100 mV to +100 mV over 5 s) was delivered to cell one, while recording currents in cell one (I1, upper traces) and cell two (current recorded in cell two while delivering voltage to cell one is equivalent, but opposite in polarity, to junctional current and is labeled Ij). Junctional current from IWCA pairs (lower left trace) are large and show non-linearity with respect to voltage that is consistent with voltage dependence of Cx43 channels. Junctional current from IKOCA pair is quite small, indicating that cells are not highly coupled. **(I)** Quantification of junctional conductance (gj = Ij/V) in IWCA and IKOCA cell pairs. Note significantly stronger gj in WCA compared to IKOCA (17.35 + 3.2 nS, *N* = 17 vs 0.36 + 0.18 nS, *N* = 23; *p* < 0.0001).

Our primary goal with the immortalization procedure was to obtain both normal astrocytes and astrocytes lacking expression of the major GJ protein Cx43, which would provide an ideal tool to assess the role of Cx43 in multiple aspects of astrocyte behavior. To assure that functional Cx43 expression persisted in the immortalized astrocytes over multiple passages, we measured intercellular transfer of the small fluorescent dyes LY (MW 444 Da) and calcein (MW 622 Da). To quantify strength of coupling with LY, we injected individual cells in confluent culture dishes. As shown in Figure 4A, at passage 9–10 LY transfer was common between IWCA, spreading from the injected cell to 13.7 ± 2.8% of its neighboring cells (Figure 4C) in a total of 535 ± 100 cells per field. LY transfer in IKOCA cultures was virtually absent (Figure 4B), spreading from the injected cell to only 1.0 ± 0.1% of its neighboring cells (Figure 4C) in a total of 102 ± 27 cells per field. Dye transfer was also evaluated using the so-called “parachute assay.” In this assay, dissociated donor cells of passage 7–10 loaded with both the GJ permeable dye calcein and the lipophilic, GJ impermeable membrane dye DiI, were added dropwise onto the monolayer of recipient cells at passage 7–10. Coupling was observed at 1–2 h after adding the donor cells. As shown in Figures 4D,G, each donor IWCA cell recruited an average of neighboring 24.36 ± 8.95 cells in IWCA recipient cultures, confirming the findings from the LY injection assay demonstrating that IWCA are functionally coupled. Heterocellular coupling between IWCA and IKOCA was not observed (Figures 4E,G), and the Cx43 deficient IKOCAs did not display homocellular coupling (Figures 4F,G).

Electrophysiological recording using whole cell voltage clamp methods provides the most sensitive and quantitative method to measure GJ mediated coupling, and the biophysical properties thus revealed can indicate which connexins form the channels (del Corsso et al., 2006). Paired whole cell recordings from IWCA (Passage >10) in response to long voltages pulses showed junctional currents that were constant over time when voltage of either polarity was low, but current declined at voltage pulses higher than about ±40 mV (Figure 4H). This weak but apparent voltage sensitivity of junctional conductance is similar to that measured in cell lines transfected with Cx43 (Moreno et al., 1995). In IKOCAs (Passage >10), fewer cell pairs were coupled and junctional conductance was much lower (Figure 4I). Calculated junctional conductance in IWCA was 17.5 + 3.2 nS in 17 cell pairs, compared to 0.36 + 0.18 nS in 23 IKOCA cell pairs, a substantial and significant difference (*p* < 0.0001).

**FIGURE 5:**
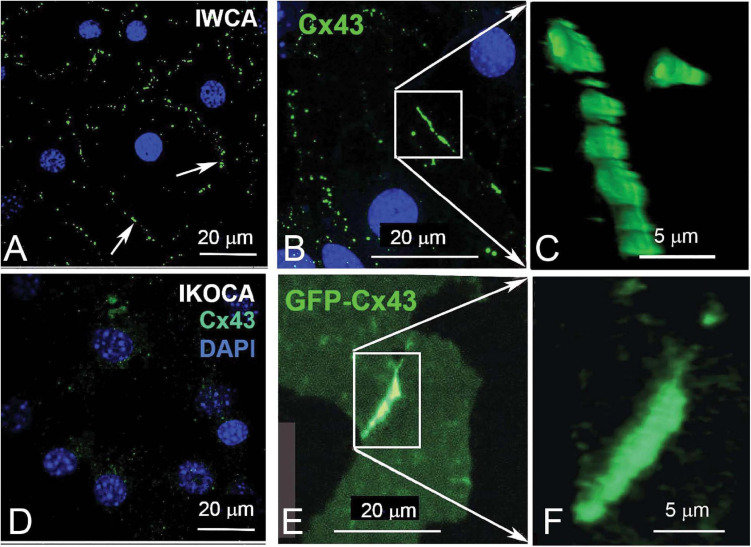
Comparison of endogenous Cx43 GJ plaques in immortalized WT cortical astrocytes (IWCA) with GJ plaques formed by GFP-tagged Cx43 in immortalized Cx43-null cortical astrocytes (IKOCA). **(A, B)** GJ plaques (arrows) outline individual IWCAs (identified by DAPI-stained nuclei). Scale bar = 20 μm. **(C)** At higher magnification, these junctional domains appear somewhat oblate, and are formed by closely packed plaques. Scale bar = 5 μm. **(D)** In IKOCAs, endogenous Cx43 junctions are absent. **(E)** Transfection of IKOCA cells with GFP-tagged Cx43 induces formation of tagged gap junction plaques. Scale bar = 20 μm. **(F)** A higher magnification image from “E.” Scale bar = 5 μm.

Findings from the dye coupling and electrophysiological studies demonstrate the preserved ability of IWCA to form functional Cx43 GJ channels. In the IKOCA, the inability to form functional Cx43 GJs is expected to be solely attributable to the lack of Cx43 expression. To demonstrate that the cellular machinery needed to assemble and form Cx43 GJs is also preserved in IKOCA, we transfected these cells with GFP-tagged Cx43 and compared GJ plaque formation with that of IWCA. As shown in Figure 5A, GJs outline the margins of IWCAs but are totally absent in non-transfected IKOCAs (Figure 5D). In IWCA, the endogenous Cx43 GJ plaques appear to consist of somewhat discrete smaller plaques when viewed at higher magnification (Figures 5B,C). Similarly, Cx43 plaques in IKOCAs transfected with GFP-tagged Cx43 appear as oblate domains containing circular subunits (Figures 5E,F). The areas of the cell in Figure 5E with dimmer but detectable GFP signal represent non-junctional sfGFP-Cx43 that can be recognized as localized mostly to the plasma membrane when viewed in 3D at high magnification using a confocal microscope. Overall increased expression of Cx43 and larger GJ plaque structures represent differences noticeable for exogenously expressed connexins and endogenously expressed Cx43 found in IWCA astrocytes. These results indicate that immortalization of Cx43-null astrocytes did not impair the ability of these cells to form GJs, and that the organization of GJ plaques in IKOCA transfected with GFP-tagged Cx43 resemble the GJ plaques endogenously expressed between IWCA. Most importantly, this finding indicates that the IKOCA cell line may be a useful substrate in which Cx43 GJs and their variants can be expressed in an astrocyte background to emulate a normal cellular context in order to investigate effects of Cx43 variant-expression on cell physiology and morphology absent the confound of endogenous Cx43 expression.

### IKOCA Cells Allow Super-Resolution Imaging of Gap Junction Plaque Morphology and Interactions With Other Organelles

**FIGURE 6:**
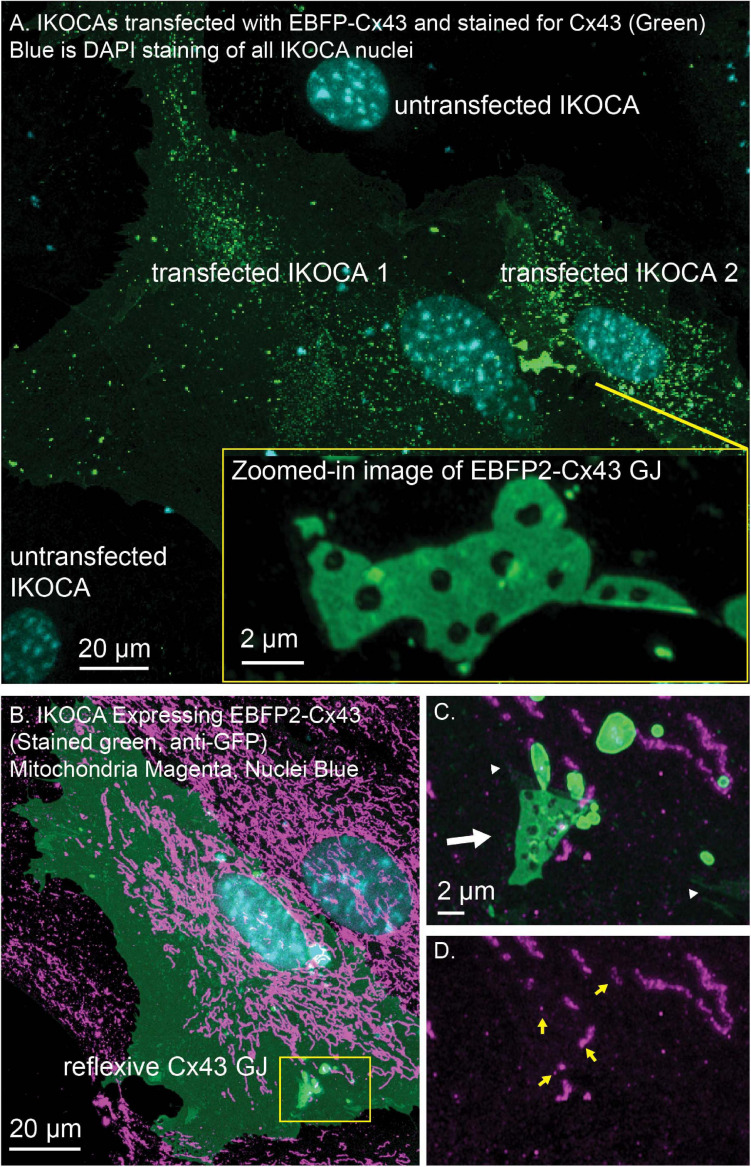
IWCA and IKOCA cells are useful to study GJ morphology and interactions with other proteins with super-resolution microscopy. **(A)** IKOCA passage 10 cells transfected to re-express Cx43 with a fluorescent protein tag (green, immunolabeling with an antibody against Cx43) form GJs when two transfected cells are in contact. As indicated by blue DAPI staining, there are untransfected cells in contact with the two transfected IKOCA cells in the center of the field of the image in A that do not express Cx43 and do not form GJs (scale bar = 20 μm). Super-resolution imaging allows insights into structural features of the GJ plaque and endocytosed GJ plaque as shown in the zoomed inset (scale bar = 2 μm). **(B)** Re-expression of fluorescent protein tagged Cx43 in a subset of IKOCA cells with subsequent immunostaining for secondary cellular features present in all IKOCA cells (e.g., mitochondria) allows investigation of the effects of Cx43 expression in astrocyte-like cells within the same cell culture sample- facilitating side-by-side comparisons and visualization of interactions between Cx43 and other organelles with super-resolution microscopy (scale bar = 20 μm). **(C)** Zoomed in view of the location where a Cx43 GJ plaque structure (green, indicated by white arrow) appears to interact with small mitochondria (indicated by TOMM20 staining, magenta). This GJ plaque represents a reflexive GJ plaque that has formed where the plasma membrane of the same astrocyte overlaps to allow GJ formation. **(D)** View of the same region as in “**C**.” with the Cx43 channel removed to allow visualization of the TOMM20 staining within endocytic Cx43 connexosomes (yellow arrows) – raising the possibility that mitochondrial transfer may occur via Cx43 endocytosis. Scale bar in **(C,D)** = 2 μm.

The locations of EBFP2-Cx43 (immunostained green in Figure 6) vesicles (green puncta) and GJ plaques connecting two IKOCA cells were imaged using a Zeiss 980 Airyscan2 microscope to examine of GJ plaque morphological features such as discontinuities and small neighboring GJ plaques (Zoomed inset in Figure 6A). Note the small bright puncta near the edge of the GJ plaque shown in Figure 6A, inset in which morphology is reminiscent of budding endocytic GJs observed previously via electron microscopy and super-resolution light microscopy by others in transformed cell lines (Bell et al., 2018). The interactions between GJs and mitochondria and with other organelles modify intercellular communication (Wang et al., 2013; Kang et al., 2014). The re-expression of Cx43 in some IKOCA cells within a culture as shown Figure 6 allows comparison of the effect of Cx43 expression in IKOCA cells on mitochondrial location and morphology. Here we show post-fixation immunofluorescence staining of all mitochondria. Note the conspicuous localization of mitochondria to areas of the EBFP2-Cx43 plaque (Figure 6C shows a reflexive Cx43 GJ plaque, white arrow) that is partially undergoing endocytosis with small mitochondria potentially located within the large endocytic portions of the GJ plaque (GJ endosomes also known as connexosomes). Connexosome-associated mitochondria are indicated by yellow arrows in Figure 6D. Areas where the astrocyte membrane is folded but not occupied by GJ plaque is indicated by the faint but enhanced green signal next to the small white arrowheads in Figure 6C. These figures highlight the utility of IKOCA and IWCA astrocytes for high resolution imaging.

### IKOCA Cells Allow Optical Experimentation on GJs and Interacting Proteins

**FIGURE 7:**
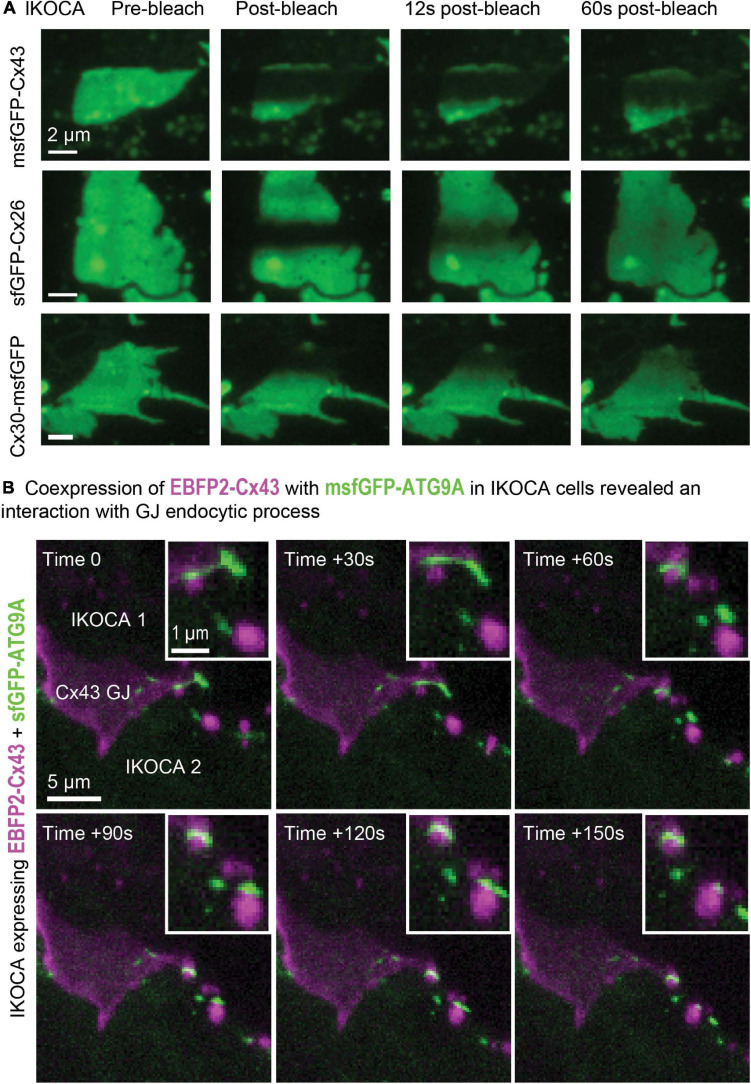
Expression of modified Cx43 transgenes in IKOCA cells can be visualized live without the confounding effects of endogenous Cx43 expression and allows testing the effects of expression of Cx43 mutants and other treatments on IWCAs. **(A)** Expression of sfGFP-Cx43 and other fluorescent protein-tagged connexins allows 3D time lapse FRAP experiments to examine GJ plaque dynamics in astrocyte-like cells (IKOCA passage 18). The thin morphology of astrocytes facilitates 3D, time lapse FRAP since GJ plaques often form in a nearly parallel configuration with the imaging focal plane. For the example images shown, the full vertical width of the GJ was imaged using 11 confocal planes with each stack acquired at 3 s intervals. The resulting Z-stacks were collapsed to a series of maximum projection reconstructions to visualize the fluorescence recovery for the entire plaque structure over time. Scale bar = 2 μm. **(B)** Expression of EBFP2-Cx43 with green fluorescent protein tags and sensors allows live, 4D examination of interactions between Cx43 and other cell components such as msfGFP-ATG9A displayed as maximum-projections as a time-lapse montage (IKOCA passage 16, scale bar = 5 μm). Please note that a EBFP2-Cx43 GJ plaque is shown in magenta with the msfGFP-ATG9A autophagy protein shown in green. The zoomed inset shows the detail of the dynamic interaction between transgene-expressed Cx43 and ATG9A in IKOCA astrocytes. Inset scale bar = 1 μm.

Three-dimensional FRAP such as that shown in Figure 7A allows improved assessment of intra-GJ plaque protein mobility since the entire effective (for this time-scale) fluorescence pool available to contribute to recovery can be detected in 3D FRAP. The motion of other GJ features such as discontinuities that form when part of the GJ is endocytosed can be easily studied in immortalized astrocytes using a confocal microscope. The arrangement of Cx43 is much more stable within the GJ plaque structure than other proteins such as Cx30 and Cx26, as reported previously in transformed cell lines (Stout et al., 2015). The thin, flat morphology of the cultured IKOCA and IWCA cells leads them to produce GJ plaques that often align nearly parallel with the growth substrate (coverslip). Such characteristics make IKOCA and IWCA cells ideal for live 3D microscopy and super-resolution imaging of GJs and intracellular organelles. Ability to entirely replace the absent Cx43 expression in IKOCA cells with fluorescent protein tagged Cx43 allows co-expression of other proteins of interest tagged with fluorescent proteins in compatible color channels. Figure 7B shows an orthogonal maximum intensity projection of a 3D time-lapse image of an pair of IKOCA cells joined by a blue-fluorescent (pseudo colored magenta) EBFP2-Cx43 GJ plaque that is partially undergoing endocytosis. The cells were co-transfected with msfGFP-ATG9A (green). ATG9A was previously shown to interact with the endocytosis process of Cx43 and to be colocalized to the plasma membrane (Bejarano et al., 2014) but the apparent transfer of small vesicle-like structures positive for ATG9A to portions of the Cx43 GJ plaque at the point of endocytosis initiation (Figure 7B, zoomed inset) was not previously reported and would be very difficult to observe in astrocyte like cells without access to IKOCA cells.

### Barrier Formation in Endothelial Monolayer Is Enhanced by Co-Culture With IWCAs

**FIGURE 8:**
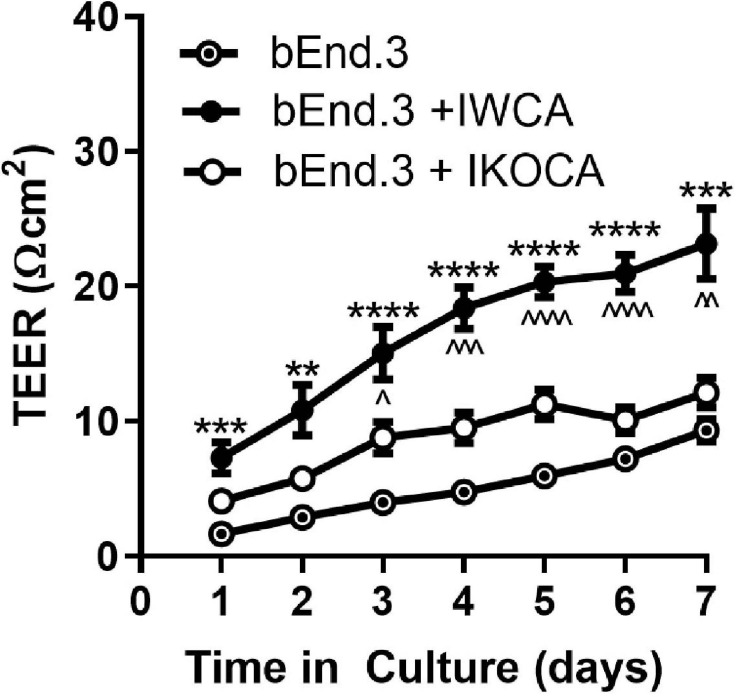
Transwell resistance (TEER) measured in bEnd.3 endothelial cells alone and in co-culture with immortalized WT (IWCA) and with Cx43-null (IKOCA) cortical astrocytes. Increase in electrical resistance of the transwell cultures of bEnd.3 cells alone and in co-culture with IWCA and IKOCA measured using the CellZscope. *N* = 11–17 separate experiments. Passage number ranged from 15–30. ^∗∗^*p* < 0.01, ^∗∗∗^*p* < 0.001, ^****^p < 0.0001 indicates significance vs bEnd.3 cells alone. ^∧^*p* < 0.05, ^∧∧^*p* < 0.01, ^∧∧∧^*p* < 0.001, ^∧∧∧∧^*p* < 0.0001 indicates significance vs bEnd.3 + IKOCA. Mixed effects analysis was used to test significance at each timepoint.

To evaluate whether the immortalized astrocyte cell lines retain their ability to functionally interact with endothelial cells, as observed at the level of the blood-brain barrier, we conducted studies with IWCA co-cultured with endothelial bEnd.3 cells. Barrier formation was evaluated based on development of Trans Endothelial Electrical Resistance (TEER) that was monitored for several days using the CellZscope apparatus. As seen in Figure 8, the TEER of cultures of bEnd.3 endothelial cells with and without IWCAs increased with time in culture. However, TEER of cultures of bEnd.3 cells with IWCA was significantly higher (*p*-value < 0.05) than either bEnd.3 alone or together with IKOCA since the beginning of the measurement and persisting until day 7. From day 1 onward, the TEER (Ohm cm^2^) of co-cultures with IWCA was significantly higher than bEnd.3 cells alone (7.31 ± 1.13 vs 1.67 ± 0.28, *p* = 0.00024) and IKOCA with bEnd.3 (7.31 ± 1.13 vs 4.33 ± 0.63, *p* = 0.033). On day 7 the difference persisted when compared with bEnd.3 cells alone (23.17 ± 2.60 vs 9.34 ± 0.86, *p* = 0.00027) and with IKOCA with bEnd.3 (23.17 ± 2.60 vs 12.56 ± 1.13, *p* = 0.0022). These IWCA co-culture TEER values are similar to those previously reported by others at 5 days from co-cultures of bEnd.3 cells with primary rat astrocytes (Li et al., 2010) using the Endohm chamber connected to an EVOM resistance meter (Srinivasan et al., 2015).

## Discussion

The immortalized cell lines that we have developed express biomarkers consistent with that of the astrocyte phenotype. In the IWCA lines, Cx43 mRNA level was rather constant during ten passages, although protein level declined slightly at passage 10 (Figure 1A). Cx43 was absent in the immortalized null line. Cx30 was absent in the immortalized cells of both genotypes (Figure 1B); this is consistent with its very low expression in neonatal astrocytes, where it only becomes detectable in older brains and in astrocytes cultured for long periods (Kunzelmann et al., 1999). Cx26, by contrast, was expressed at moderately high levels in early cultures and declined with passage. Likewise, the water channel that characterizes the astrocyte endfoot, AQP4, was expressed at highest level in early passages and its mRNA and protein declined to very low levels by passage 10 (Figure 1D). As discussed below, such decline in astrocyte-specific protein expression may be caused by lack of the reciprocal signaling between astrocytes and other brain cell types that occurs in the intact brain. The mRNAs and protein levels of four astrocytic biomarkers, ALDH1L1, GS, GLT-1, and GFAP were also assessed. ALDH1L1 and GS mRNAs were relatively constant during successive passages (Figures 1E,F), whereas GS protein levels actually increased until passage 10 (Figure 3D). Expression of glutamate transporter GLT-1 and GFAP mRNA declined with passage; GFAP protein level declined but was still detectable at passage 10 (Figures 2G,H, 3C). Immunostaining at corresponding times substantiated the Western blots and also indicated that expression was in appropriate compartments within the cells.

Gap junction mediated intercellular communication was maintained in the IWCA line and was very low in the IKOCA line. With respect to transfer of small molecular weight dyes, both intracellularly injected LY and ester loaded calcein were highly permeable to junctions between cells. Electrophysiological measurements of electrical coupling found junctional conductance averaging 17 nS between IWCA cell pairs, which is similar to the value of 13 nS that we have previously reported in primary cultures of these cells (Dermietzel et al., 2000). Voltage sensitivity of junctional conductance, a distinctive biophysical property of GJs formed of each connexin, was similar to that reported for Cx43 (Dermietzel et al., 2000).

The development of methods that permitted mammalian cells to be maintained in culture for prolonged periods of time was a major accomplishment that pioneered the fields of cell biology and cellular neuroscience. Cell types from some tissues continue to be much more difficult to extract and maintain in culture than oncogenically transformed cell lines. Most differentiated primary cells extracted from tissue undergo a limited number of cell divisions before becoming senescent, necessitating continuous cell harvesting to maintain a supply of material dissociated from tissues for study. The most commonly used transformed cell lines have contributed greatly to cell biological research but have a number of well-known drawbacks including major changes to cell phenotypes, signaling pathways and characteristics of intercellular interactions. It has thus been desirable to obtain cell lines that divide continuously and which also express phenotypes resembling the cell type of interest.

Conventional methods to obtain immortalized cell lines have involved the isolation of cells from tumors [see (Souza et al., 2016), examples of lines initially generated (earlier than 1960) being a mouse lung cell line (L1), the highly discussed and widely used human cervical cell line [HeLa; (Lucey et al., 2009)] and Chinese hamster ovary cells (CHO)], but thousands of other cell lines have since been derived from tumors and characterized to some extent or another. It is now known that these cell lines arose from oncogene-expressing cells. HeLa cells are infected with human papillomavirus (HPV), which synthesizes proteins that inactivate the tumor suppressor molecule p53 and others (Lechner et al., 1992).

Studies of mechanisms responsible for tumorigenesis have exploited SV40, a polyomavirus that was discovered in a monkey cell line used to produce poliovirus vaccine. SV40 infects monkey cells, activating expression of early and late genes, and does not produce tumors in monkeys. In rodent cells, SV40 generates both large and small T-antigens to inhibit pRb and p53 as well as a protein phosphatase (PP2A) (Ahuja et al., 2005). In mice, SV40 virus infection leads to the induction of tumors and has been used to generate a number of widely used mouse cell lines [reviewed in Hudson and Colvin (2016)]. In cell culture, SV40 infection leads to cell transformation, generally assayed as loss of contact inhibition of growth, ability to grow in soft agar and decreased requirement of serum for survival. Most cell lines in current use were generated by this method and thus exhibit the transformed phenotype. For example, the American Tissue Cell Collection repository contains seven astrocyte cell lines. One of these DI TNC-1, obtained by GFAP-targeted oncogene delivery to neonatal rat diencephalon astrocytes (Radany et al., 1992), has low levels of endogenous aquaporins and has thus been a useful model to study exogenous constructs expressed in an astrocyte-similar transformed cell line [e.g., (Mola et al., 2016)]. Products are even commercially available tailored specifically to such studies^1^.

A more recent alternative for the immortalization of primary cells involves the incorporation of hTERT, which preserves length of telomeres and thus endows cell with properties of self-renewal. hTERT introduction has an advantage over the use of viral protein for immortalization in that it does not involve the inactivation of tumor suppressor gene, thereby bypassing the transformed phenotype (Kogan et al., 2006). Thus, hTERT immortalization results in cells that typically more closely resemble the primary cells than do virus-transformed cells, although both immortalization methods escape the limited number of cell cycles before senescence and may not totally mimic the properties of the cells from which they are derived.

Most studies using cell lines to evaluate consequences of GJ expression on astrocyte phenotype have used C6 glioma cells (ATCC #CCL-107). This cell line was derived from rat brain tumors generated by nitrosomethylurea exposure and was initially characterized as rich in S100 protein and spindle-shaped (Benda et al., 1968). The C6 cell line is widely used in studies of glioblastoma invasion, as it forms robust tumors *in vivo*. It has been described as containing two populations of cells, one having markers of immature oligodendrocytes and another being a mixture of oligodendrocyte and immature astrocytes (Coyle, 1995). Notably, GFAP expression is absent and vimentin expression is variable in C6 glioma cells (Chou et al., 2003). With regard to GJ research, C6 glioma cells were found to express less Cx43 than astrocytes, and are regarded as being severely communication deficient (Naus et al., 1991). In a number of studies, overexpression of Cx43 in these cells has been reported to decrease tumor growth rate *in vivo* and cell proliferation in culture [see (Zhu et al., 1992)]. However, C6 cells form functional GJ with astrocytes (Zhang et al., 1999), and numerous studies have more recently indicated that GJ communication though Cx43 channels plays a major role in glioblastoma invasion [see (Umans and Sontheimer, 2018)]. Opposite effects were observed for primary mouse astrocyte cultures (with decreased proliferation for astrocytes extracted from Cx43-null mice) (Naus et al., 1997; Dermietzel et al., 2000). The multiple lines (with somewhat varied expression of other astrocyte proteins) of paired Cx43-null and WT astrocytes that we report here will allow clarification of these intriguing findings relating Cx43 expression on astrocyte proliferation because mutant forms of Cx43 can be re-expressed in the IKOCA cells (as shown in Figures 5–7) to dissect the mechanistic basis of Cx43 effects on cell growth, particularly in a fully Cx43-null background without potential off-target effects that are inherent to current genome editing techniques.

Few studies directly related to the issue of tumorigenesis have been conducted on astrocytes derived from Cx43 deficient astrocytes. However, it is clear that manipulation of Cx43 expression in C6 cells may have very different consequences than in astrocytes. For example, in a study comparing gene expression levels in WT C6 cells with three Cx43 transfected clones, only seven distinct genes were found to be up or downregulated (Naus et al., 2000). In transcriptome-wide studies comparing astrocytes from WT mice with either astrocytes from Cx43-null mice (Iacobas et al., 2003) or following Cx43 knockdown with siRNA (Iacobas et al., 2008), hundreds of genes were up or downregulated. Gene regulation in Cx43-null and Cx43 knockdown astrocytes was to some extent similar (Iacobas et al., 2008), but none of the genes identified in the transfected C6 cells was regulated in either of the Cx43-null or Cx43 knockdown astrocytes. The use of hTERT to immortalize the astrocyte lines we describe is expected to have less side-effects than oncogene-induced transformation on cell signaling networks, but it should be noted that Park et al. (2009) reported that hTERT expression can modulate the Wnt pathway. A promising area for future studies could examine the effect of the hTERT transgene and exogenous expression on the Wnt and downstream pathways through LoxP or CRISPR-Cas9 mediated excision of the transgene used in the IWCA and IKOCA cells. Importantly, the multiple cell lines characterized in this study and ability to re-express Cx43 will allow for more reliable studies through multiple lines and control for expression of Cx43 on other cell pathways [there are strong reciprocal interactions between Cx43 and Wnt pathways (van der Heyden et al., 1998; Ai et al., 2000; Hou et al., 2019; Lopez et al., 2019)].

The cell lines developed here exhibit characteristics of primary astrocytes and are expected to offer new opportunities for the evaluation of the roles of astrocyte GJs than cells derived from glioblastomas or other tumors. For example, future studies examining binding partners and their affinity for astrocyte proteins (McCutcheon et al., 2020b) are likely to be more similar to those that occur *in vivo* when examined in a more natural context (although the presence of Cx26 expression in IKOCA cultures should be considered). It also has to be considered, that we found substantial variability across the immortalized astrocyte cell lines, between Cx43 genotype, and across passage number with regard to in expression of some proteins that are known to be expressed in astrocytes *in vivo*. This finding is not surprising in light of the substantial gene and protein expression level-differences observed between astrocytes in primary cell culture compared with *in vivo* expression levels. Additionally, there is known heterogeneity in astrocyte gene expression during development, between cortical subregions, and even across layers of the same region of the cerebral cortex (Batiuk et al., 2020) and as reviewed by several groups (Schitine et al., 2015; Farmer and Murai, 2017; Westergard and Rothstein, 2020). Significant differences between *in vivo* astrocyte gene expression make the finding of substantial variability in gene expression levels in immortalized astrocytes cultured from the entire cortical astrocyte population unsurprising, since the cells undergo immortalization which represents bottleneck in the cultured cell population. The variability between immortalized astrocyte lines across passage number could be caused by differences in growth factor signaling and altered extracellular environment for cultured cells versus the *in vivo* conditions where other brain cell types have been found to profoundly alter astrocyte gene-protein expression (Hasel et al., 2017). Expression of GLT-1 in cultured astrocytes was shown to depend on neurons and endothelial cells (Martinez-Lozada and Robinson, 2020). Therefore, the low or undetectable GLT-1 expression is expected for isolated immortalized astrocyte cultures. Whether co-culture of immortalized astrocytes with neurons and brain endothelial cells can induce GLT-1 expression in hTERT immortalized astrocytes represents an exciting area for future research. Additionally, the influence of the presence or lack of Cx43 could be examined and we anticipate differences in GLT-1 localization based on experimental results when Cx43 and glutamate transporters were over-expressed in non-astrocyte cell lines (McCutcheon et al., 2020b). Changes in protein expression over passage number are also expected to occur by the numerous mechanisms that produce substantial differences in genotype and phenotype in widely used transformed cell lines, such as HeLa cells and other high-passage cell lines. We show data in Figures 6, 8 in which cells were used with passages further than those we characterized in this study. Future studies that examine the effect of astrocytes with and without Cx43 expression on autophagy and paracellular tight junction promotion will need to verify Cx43 expression or lack of expression and other astrocyte markers, but the results presented in these figures do indicate that these cells can be used to higher passage numbers without encountering senescence. The hTERT-derived lack of senescence means the immortalized astrocytes we developed and describe in this report may not be suitable for studies on astrocyte physiology during aging and development. In addition to the effect of endothelial cell expression on astrocyte expression, the finding in Figures 1, 2 indicating decreasing expression of AQP4 across passage number, mean that verification of AQP4 expression level will be important for future use of the immortalized astrocyte lines described here. Future studies that examine the effect of Cx43 expression and functional outcomes will need to test the level and cellular localization of AQP4 because a direct interaction between Cx43 and AQP4 has been shown in past research (Rash and Yasumura, 1999; Nicchia et al., 2005; Katoozi et al., 2017, 2020). Prior studies have shown that IL-1β, IL-1α, NFκB, and other factors produce important changes to gene expression, morphology and GJ connectivity *in vivo* and in primary astrocyte cultures. The response of immortalized astrocytes and effect of Cx43 expression under inflammatory and cell injury conditions is another exciting application for IWCA and IKOCA astrocytes in future studies.

The aforementioned areas of investigation are some among several others that can benefit from use of the WT and Cx43-null immortalized cell lines described here. The degree to which properties of astrocytes and their responses to physiological and pathological conditions depend on Cx43 expression, and identification of responsible molecular mechanisms and domains should be readily accessible and facilitated with the use of these astrocyte cell lines derived from sibling WT and Cx43-null mice.

## Data Availability Statement

The raw data supporting the conclusions of this article will be made available by the authors, without undue reservation.

## Ethics Statement

The animal study was reviewed and approved by the Institutional Animal Care and Use Committee (IACUC) at Albert Einstein College of Medicine.

## Author Contributions

All authors listed have made a substantial, direct and intellectual contribution to the work, and approved it for publication.

## Conflict of Interest

The authors declare that the research was conducted in the absence of any commercial or financial relationships that could be construed as a potential conflict of interest.

## Footnotes

1 https://altogen.com/product/di-tnc1-transfection-reagent-rat-brain-astrocytes/

## Abbreviations

ALDH1L1: 10-formyltetrahydrofolate dehydrogenase
DiI: 1,1 ′ -dioctadecyl-3,3,3 ′ 3 ′ -tetramethylindocarbocyanine perchlorate
AQP4: Aquaporin4
bEnd.3 cells: brain microvascular endothelial cell line from mouse ATCC^®^ CRL-2299 ^TM^
Cx43: Connexin43
Cx30: Connexin30
Cx26: Connexin26
EBFP2: enhanced blue fluorescent protein 2
FRAP: Fluorescence Recovery After Photobleach
GS: glutamine synthetase
GFAP: Glial Acidic Fibrillary Protein
GJ, GLT-1 (EAAT2): Glutamate transporter 1 (Excitatory Amino Acid Transporter 2): Gap Junction
hTERT: human telomerase reverse transcriptase
IWCA: immortalized wildtype cortical astrocytes
IKOCA: immortalized Cx43-null cortical astrocytes
msfGFP: monomerized superfolder green fluorescent protein
qRT-PCR: quantitative real-time polymerase chain reaction
sfGFP: superfolder green fluorescent protein.
